# Automated Calibration of a Poly(oxymethylene) Dimethyl Ether Oxidation Mechanism Using the Knowledge Graph Technology

**DOI:** 10.1021/acs.jcim.0c01322

**Published:** 2021-04-07

**Authors:** Jiaru Bai, Rory Geeson, Feroz Farazi, Sebastian Mosbach, Jethro Akroyd, Eric J. Bringley, Markus Kraft

**Affiliations:** † Department of Chemical Engineering and Biotechnology, University of Cambridge, Philippa Fawcett Drive, Cambridge CB3 0AS, U.K; ‡ Department of Computer Science and Technology, University of Cambridge, 15 JJ Thomson Avenue, Cambridge CB3 0FD, U.K.; § Cambridge Centre for Advanced Research and Education in Singapore (CARES), CREATE Tower #05-05, 1 Create Way, Singapore 138602, Singapore; ∥ School of Chemical and Biomedical Engineering, Nanyang Technological University, 62 Nanyang Drive, Singapore 637459, Singapore

## Abstract

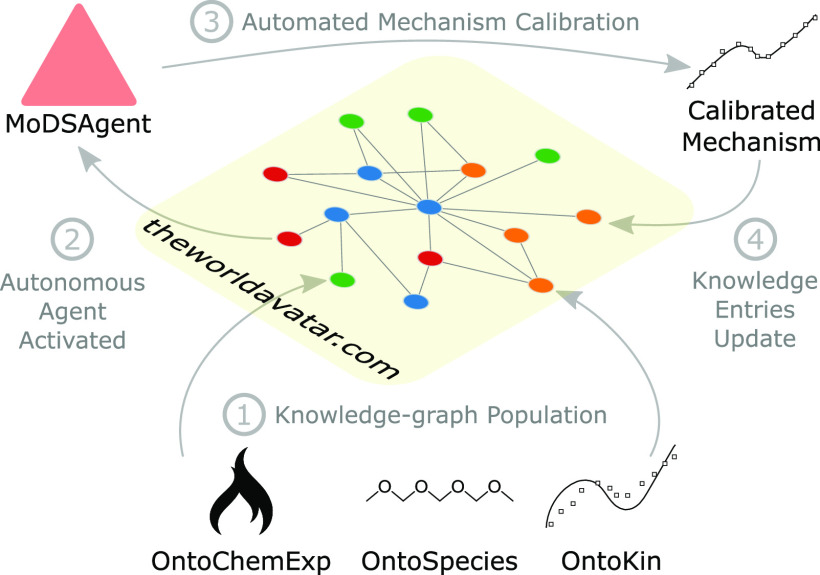

In this paper, we develop a knowledge graph-based framework for the automated calibration of combustion reaction mechanisms and demonstrate its effectiveness on a case study of poly(oxymethylene)dimethyl ether (PODE_*n*_, where *n* = 3) oxidation. We develop an ontological representation for combustion experiments, OntoChemExp, that allows for the semantic enrichment of experiments within the J-Park simulator (JPS, theworldavatar.com), an existing cross-domain knowledge graph. OntoChemExp is fully capable of supporting experimental results in the Process Informatics Model (PrIMe) database. Following this, a set of software agents are developed to perform experimental result retrieval, sensitivity analysis, and calibration tasks. The sensitivity analysis agent is used for both generic sensitivity analyses and reaction selection for subsequent calibration. The calibration process is performed as a sampling task, followed by an optimization task. The agents are designed for use with generic models but are demonstrated with ignition delay time and laminar flame speed simulations. We find that calibration times are reduced, while accuracy is increased compared to manual calibration, achieving a 79% decrease in the objective function value, as defined in this study. Further, we demonstrate how this workflow is implemented as an extension of the JPS.

## Introduction

The contribution of human activity to climate change and the potential for ecological devastation this presents has become a widely accepted fact within the scientific community.^1^ One of the key contributors to this effect is the release of greenhouse gases from combustion processes. Improvements in the design of the energy conversion system has already resulted in significant reductions in their contribution to greenhouse gas emissions and presents one of the potential paths toward even lower emissions in the future. Another approach involves the use of alternative fuels, particularly synthetic fuels, offering potential for reductions to pollution and greenhouse gas emissions.

Modern workflows for the design and optimization of combustion equipment and devices now routinely employ computational modeling techniques. These are most often used to screen designs and offer invaluable insights into processes occurring within ref (2). Thus, to achieve the desired reduction in climate change potential from combustion equipment, provision of accurate combustion chemistry mechanisms is becoming essential.

In practice, the development of these combustion chemical mechanisms consists of two parts: mechanism generation and mechanism calibration. The first step constructs a tentative mechanism that maps the reaction pathways via elementary reaction generation and selection, and the second step adjusts the rate parameters, attempting to faithfully reproduce experimental observations.

Much of the construction of combustion mechanisms involves the selection and combination of reaction families, elementary reactions, and submechanisms from various existing mechanisms. This is facilitated by the CHEMKIN^3^ mechanism format, acting as a *de facto* standard for mechanism sharing within the combustion community. Aspects not formally captured by this format include semantics and provenance. This allows errors to propagate through combustion models due to the inability to ensure the quality of individual reactions and the difficulty of tracking their origin.

Moreover, accuracy and consistency of combustion mechanisms is not guaranteed across applications, even with well-calibrated submechanisms.^4^ These problems are further exacerbated when the scale of the mechanisms is considered; potentially, hundreds of species and thousands of reactions may be involved. This leads to the manual curation and provenance determination of all the components of these mechanisms being a near-impossible task for researchers. Even if attempts were to be made, these are likely to fall foul to human error, and so a very real need for an automated approach to this mechanism development task is present.

Reaction mechanism generator (RMG)^5^ is one of the available tools for the automation of the first stage of mechanism development. The technique is based on the use of a set of chemical rules to predict chemical pathways along with a database of chemical properties. Values of unknown chemical properties are estimated on-the-fly. One of the methods used for this purpose is that of Li et al.,^6^ employing a graph neural network (GNN) on molecular graphs to make formation enthalpy predictions. The GNN was trained on a data set generated at the B3LYP/6-31G(2df,p) level of density functional theory (DFT). The framework further incorporated quantum chemistry calculations for additional model training in case of uncertain predictions, improving accuracy and generalizability.

Progress has also been made in automating transition-state theory calculations,^7^ an important step toward generating accurate reaction rates. However, generating a chemical mechanism with purely quantum calculated rate parameters remains infeasible, given that even the most detailed model would not include all possible pathways.^1^ This necessitates the use of automated calibration processes for these coefficients to reproduce experimental results.

The mechanism development and curation process may be improved in two ways: (1) semantically focused and machine-interpretable formats for mechanism representation with clear provenance should be used and (2) automated updating and verification of existing mechanisms throughout their lifetimes should be implemented. Task 1 has received attention within the community, with various efforts to create standardized databases of combustion data with unique identifiers and easily processable formatting. One of the key efforts in this direction is that of the Process Informatics Model (PrIMe)^8^ database, containing combustion data in a standardized eXtensible Markup Language (XML) format. Varga et al.^9^ further developed the ReSpecTh kinetics data (RKD) format, which is an extended version of the PrIMe XML format with new elements for unique identification of the experimental setup and data. Computational packages, for example, Optima++,^10^ are also provided alongside RKD for carrying out simulations and interpreting experimental results. ChemKED^11^ and its related Python-based package is another effort in providing a standard format for experimental data in the combustion community.

Although projects such as PrIMe have started the process, further strides toward a fully provenanced and machine-interpretable standard for the combustion community must continue. The relatively granular structure of databases and the lack of semantics prevents them from reaching the true potential of modern technologies within artificial intelligence and knowledge discovery. One of the potential technologies to aid with these processes are knowledge graphs. A knowledge graph is a dynamic knowledge ecosystem interconnecting individual pieces of information and software. This is implemented using ontologies, commonly written in the Web ontology language (OWL), to define the abstract concepts and relations that are shared within the knowledge graph. Such a design offers both clear semantics to its entries and a highly linked structure, thus enabling item location, easy provenance determination, and reasoning over entries with software agents.

In the context of combustion chemistry, we have developed OntoKin^12^ as an ontology for representing chemical kinetic reaction models. We have also developed OntoCompChem,^13^ based on chemical markup language,^14^ to store quantum chemistry calculations. We further introduced OntoSpecies^15^ for unique chemical species identification, generating a more comprehensive description of combustion chemistry with the three ontologies seamlessly linked together to enable consistency checking across multiple mechanisms.^16^ This framework was further enhanced by the development of a set of autonomous agents for quantum chemistry and enthalpy of formation calculations, employing error-canceling balanced reactions for the enthalpy of formation calculations.^17^

The purpose of this work is to propose a knowledge graph-based framework for automated combustion mechanism calibration. This forms a clear path for achieving the second of the highlighted tasks. We aim to achieve this by constructing an ontology to link combustion experiment measurements to chemical reaction mechanisms and developing a set of software agents that automatically perform mechanism calibration against ignition delay time and laminar flame speed experimental data. A demonstration of this is performed on a reduced poly(oxymethylene)dimethyl ether 3 (PODE_*n*_, where *n* = 3) mechanism.^18^ This alternative fuel, with a molecular formula of CH_3_O(CH_2_O)_3_CH_3_, is deliberately chosen for its current interest as a fuel additive to help with the push for cleaner and more efficient vehicles. The additive has been demonstrated to lower soot emissions and improve combustion efficiency in engines.^19^ To match the current interest and to further PODE_3_’s commercialization, a reduced yet robust mechanism is required, making it an ideal candidate for a demonstration of our framework.

The presentation of this paper is structured as follows. The next section situates this work by introducing the wider knowledge graph-based project. Subsequently, we detail the components that together form the framework. We then present the demonstration case of PODE_3_ with significant improvements in model performance and finally conclude the work.

## The World Avatar

“The World Avatar” (theworldavatar.com) begins the process of creating a fully interconnected virtual representation of the world through semantic web technologies. The term originated from the idea of the “Digital Twin” in Industry 4.0 but extended the “Digital Twin’s” representation of factories to conceptualizing and representing everything that physically exists. With this vision, “The World Avatar” aims to standardize the language used across knowledge domains to enable cross-domain communications, offering extensive opportunities for solving more complex and interesting problems.^20^

The J-Park simulator (JPS)^21^ is an instantiation of “The World Avatar” at the intersection of chemical and electrical engineering. The initial scope of the JPS is to create a digital replica of the ecoindustrial park on Jurong Island, Singapore.^22^ The effectiveness of JPS has been demonstrated through its ability to solve many energy related problems. The JPS has been applied to the utilization of waste energy,^23^ network optimization of the ecoindustrial park,^24^ and simulations of a carbon tax for scenario analysis in policy making.^25^

**Figure 1:**
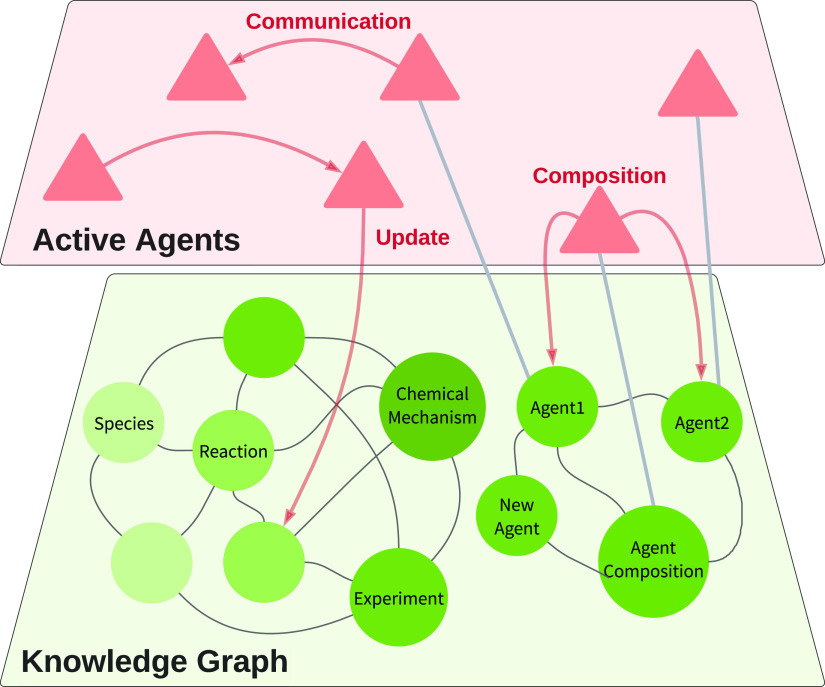
Structure of the JPS as an implementation of The World Avatar. Interoperable agents are part of the distributed knowledge graph and operate on it once they are activated.

The versatility of the JPS is a result of two key components: modular and reusable ontologies and interoperable agents. As illustrated in Figure 1, these components form the core of the JPS—a distributed knowledge graph. By design, the JPS employs both in-house ontologies developed by domain experts (e.g., OntoEIP,^26^ OntoCityGML,^21^ OntoPowSys,^27^ etc.) and existing ontologies developed by external researchers (e.g., DBpedia,^28^ OntoCAPE,^29^ etc.). These ontologies are connected and merged within the JPS to ensure the depth and breadth of the concepts and relations in the knowledge graph. Data entries from different sources are described in the languages of these ontologies and stored in decentralized locations. The data are indexed with their own internationalized resource identifiers (IRIs) that can be addressed without ambiguity.

Besides domain ontologies, the JPS employs an agent ontology (OntoAgent^30^) to govern the concepts related to agents interacting within the knowledge graph. Each agent is an individual building block, defined for specific tasks. As the agents share the same architecture and follow a similar design pattern, once activated, they are able to communicate with each other. The agents may further operate cooperatively in tasks ranging from manipulating the data within the knowledge graph to coordinating between the JPS and the outside world. To ensure secure agent operations, block chain technology was implemented to support automated agent selection with a tamper-proof agent marketplace.^31^ Once granted access privileges, these tasks are envisaged to be completed without human interventions.

The current design and implementation of the JPS has two significant advantages, namely, reusability and extensibility. In that sense, the JPS sets the standard and provides the basic toolbox, facilitating individual researchers and developers to build a customized knowledge graph. As its ecosystem expands, the JPS has access to high volumes of real-time data from the real world, enabling simulation of dynamic physical processes in the cyber space. By connecting the real world and its virtual representation, the JPS adds a new dimension of intelligent operation in the engineering sector.

The work described in this paper connects the measurements made during combustion experiments to relevant reaction mechanisms via the unique identification of chemical species. A distinguishing feature of the ontology and the agents we have developed is their seamless addition to an existing knowledge graph, in which all entities are relevantly linked. This presents a next step towards connecting macroscopic, observable data collected in real world experiments with quantum chemistry calculations. Relating experimental data to reaction mechanisms, and subsequently to individual reactions, presents a valuable step toward enhancing mechanism development with accurate chemical kinetics. This step further pushes in the direction of combining data from a wide variety of domains, scales, and sources to support cross-domain communication and scenario planning, the relevance of which is demonstrated in atmospheric pollutant dispersion simulation.^17,21^

## Methodology

### Mechanism Calibration

#### PODE Demonstration Case and Simulation Procedure

This case serves as a demonstration for the developed framework. The particular case of PODE_3_ has received recent interest and a range of previous efforts for modeling its combustion processes accurately when used as part of a fuel blend. Some of the prior works in this area are listed in Table 1, with many of the mechanisms intended for primary reference fuel (PRF) blends.

**Table 1:** Summary of Existing PODE_*n*_ (*n* = 2, 3, 4) Combustion Mechanisms with Their Statistics Counted in the OntoKin Format^a^

mechanism	type	no. species	no. reactions	fuel	fuel carrier
Sun et al.^32^	detailed	274	1674	PODE_3_	no
He et al.^33^	detailed	225	1178	PODE_3_	no
He et al.^34^	detailed	354	1392	PODE_3_	yes, PRF
Ren et al.^35^	reduced	145	668	PODE_3_	yes, PRF
Lin et al.^18^	reduced	61	215	PODE_3_	yes, PRF
Cai et al.^36^	detailed	322	1611	PODE2–4	no

a Reduced mechanism developed in Lin et al.,^18^ before optimization, is chosen as the starting mechanism for the demonstration case of the knowledge graph-based automated mechanism calibration approach proposed in this work. It should be noted that the number of reactions of the OntoKin representation is different from that of the CHEMKIN format.

The first mechanism for pure PODE_3_ combustion under high-temperature conditions was developed by Sun et al.^32^ This was an example of a detailed combustion mechanism, whereby an attempt is made to model all elementary reactions believed to be present. In contrast, reduced combustion mechanisms are constructed to replicate results of key combustion metrics with a reduced set of reactions.

Following from the Sun et al.^32^ mechanism, low- and intermediate-temperature conditions were covered by He et al.^33^ This model was further expanded by He et al.,^34^ which is the first-ever mechanism to describe the combustion of a PODE_3_/PRF blend. Given the complexity of engine simulations, the size of these mechanisms makes simulation largely intractable, requiring the development of a reduced mechanism. Two simplified mechanisms were proposed by Ren et al.^35^ and Lin et al.^18^ Both employed the model of He et al.^33^ as a basis, using different methodologies for selecting key species and reactions of PODE_3_. Additional reactions were added by both for modeling the combustion of a PRF carrier fuel.

A notable alternative attempt was made in the work of Cai et al.^36^ In this, an automated mechanism development process is used to select the reactions for the detailed combustion mechanism of PODE_*n*_ (*n* = 2, 3, 4). The work employed the class-based automatic reaction alternator and calibrated the selected reactions against experimental data for the ignition delay of PODE_*n*_/air mixtures.

As a demonstration of the proposed knowledge graph-based approach, the starting mechanism of Lin et al.^18^ is selected due to its relatively small size. This is the mechanism generated by selecting reactions from the wider He et al.^33^ mechanism prior to any further calibration to experimental results. The focus of this paper remains the calibration of the PODE_3_ combustion mechanism, and so only PODE_3_ combustion experiments are chosen for calibration.

The calibration was carried out against rapid compression machine ignition delay time^33^ and laminar flame speed^32^ experiments. The ignition delay times of PODE_3_/O_2_/N_2_ mixtures were measured at pressures of 10 bar and 15 bar, over a temperature range of 641–865 K, with equivalence ratios of 0.5 (O_2_/N_2_ = 1:8), 1.0 (O_2_/N_2_ = 1:15), and 1.5 (O_2_/N_2_ = 1:20). The laminar flame speeds of PODE_3_/air mixtures were measured at atmospheric pressure and an initial temperature of 408 K, with equivalence ratios ranging from 0.7 to 1.6. For the simulation stage, the ignition delay time is defined as the time interval between the starting point and the maximum rate of pressure rise due to the ignition. The laminar flame speeds were calculated with a mixture-averaged transport model. The simulations were performed using *k*inetics (version 2020.1.1)^37^ for ignition delay times and Cantera (version 2.4.0)^38^ for laminar flame speeds. For the laminar flame speed simulations, Soret effects were not considered and the solution gradient and curvature were both fixed at 0.05. The grid was set to be refined with a pruning coefficient of 0.01.

A core strength of a knowledge graph approach is its ability to combine data and software from different sources in a standardized way, achieving interoperability and extensibility. In the present application, this means the ability of a generic tool for calibration of any gray- or black-box model to deal with a variety of computational software. As a first step toward this goal, different modeling software for ignition delay times and laminar flame speeds are employed to demonstrate the competence of the framework in handling models in a generic manner.

#### Sensitivity Analysis

Sensitivity analysis acts as a screening process to identify reactions that have measurable effects on the model responses.^39^ This is conducted by computing the normalized sensitivity coefficient of the chosen response with respect to the Arrhenius pre-exponential factors of the starting mechanism.

At the *n*th point in the process condition space ξ^(*n*)^, the normalized sensitivity coefficient of the *i*th response η_*i*_(ξ^(*n*)^,θ) with respect to the *j*th model parameter θ_*j*_ is defined as^40^
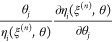
1

Due to the complexity and stiffness of a typical combustion mechanism, a numerical solution is normally adopted.^40^ The vector showing a small relative perturbation *r* of model parameters in the *j*th positive direction can be denoted as
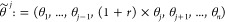
2yielding a finite difference approximation of the normalized sensitivity coefficient as
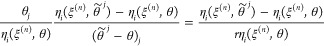
3

Considering the sensitivities across the entire range of process condition space *N*, the overall sensitivity *S*_*ij*_ of the *i*th response with respect to the *j*th model parameter can be determined either by a maximum absolute value
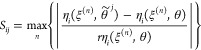
4or an averaged absolute value
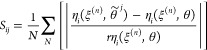
5

It should be noted that this analysis is local in the sense of model parameters while global in the process condition space such that sensitivities at every collected point in the experiment are taken into account.

As chemical mechanisms can either be assembled from reaction classes or individual elementary reactions, it is natural to either optimize reactions on a class basis or just based on the individual reaction’s contributions. Cai and Pitsch^41^ demonstrated a comparable performance between both bases, claiming that a significant distinction would only appear when reactions in the same class show low sensitivity individually but high sensitivity collectively.

In the case of a combustion mechanism constructed for a group of similar species, optimization based on reaction rules often provides a consistent improvement of model performance. This was found to be the case with the mechanism developed by Cai et al.,^36^ describing PODE_2–4_ combustion. The comparable performance is seen as justification for implementing only one of the bases at present. The selected basis is that of individual reaction contributions, chosen because many of the envisaged use cases will involve only one or few key staring species. Further options for calibration on a class basis will be implemented in future work.

#### Global Search and Local Optimization

In order to find an optimal balance between the two considered responses, a weighted least-squares objective function was implemented for the experiment responses
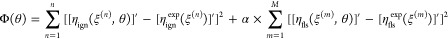
6where α refers to the weighting of laminar flame speed in the calibration process.

Following selection of the target reactions through sensitivity analysis, an optimization routine is followed to calibrate the mechanism with the objective function defined above. The process initially employs low-discrepancy quasirandom global sampling through a Sobol sequence generator.^42^ This provides initial points for a Hooke–Jeeves optimization algorithm,^43^ selected for its gradient-free nature to better handle the stiff system.

In each evaluation, experiment and model responses are scaled with respect to the upper η_ub_ and lower η_lb_ bounds of the experimental observations, as defined by the experimental uncertainty. For ignition delay times, a ±20% uncertainty was assigned to the measurements. This was selected to align with common practices within the community.^36,44,45^

As ignition delay times can vary by orders of magnitude, a logarithmic scaling was applied to balance the contribution of each data point toward the objective function
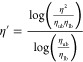
7

For laminar flame speed data, the error used was that reported by the source.^32^ A linear scaling was applied
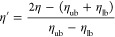
8

Uncertainty bounds may be obtained from uncertainty factors for Arrhenius rate equation parameters,^46^ derived from the uncertainties in quantum chemistry calculations. There are also alternative optimization principles for the reactions involved in combustion chemistry that optimize both activation energies and pre-exponential rate parameters in a coupled manner,^47^ as well as techniques that include the temperature-dependence exponent.

At present, the optimization of just pre-exponential factors has been performed. This was chosen to simplify the process for a proof-of-concept framework demonstration and as the main focus of this work is to demonstrate a knowledge graph-based approach. There is an existing precedent for works adjusting the pre-exponential factors alone,^18^ with large hypercubes of potential values. This approach is further justified when reduced mechanisms are optimized as some reaction pathways and species are already not present within the mechanism. It should be noted that other optimization techniques can be easily made available in future work.

### Ontological Representation

The OntoChemExp ontology is developed to describe combustion experiments, detailing both the overall experimental setup and the individual, independent variable values for each data point. The overall experimental description of the ontology incorporates the apparatus used and the various common properties shared among data points. Independents are used to form data groups that share the same set of independent variables, with individual data points forming subsets of these data groups.

The current structure of OntoChemExp is developed following discussions with domain experts and takes inspiration from existing databases, including the experimental data stored in the PrIMe database. The complete ontology contains 36 concepts and 60 relations. OntoChemExp is published at: http://www.theworldavatar.com/ontology/ontochemexp/OntoChemExp.owl.

**Figure 2:**
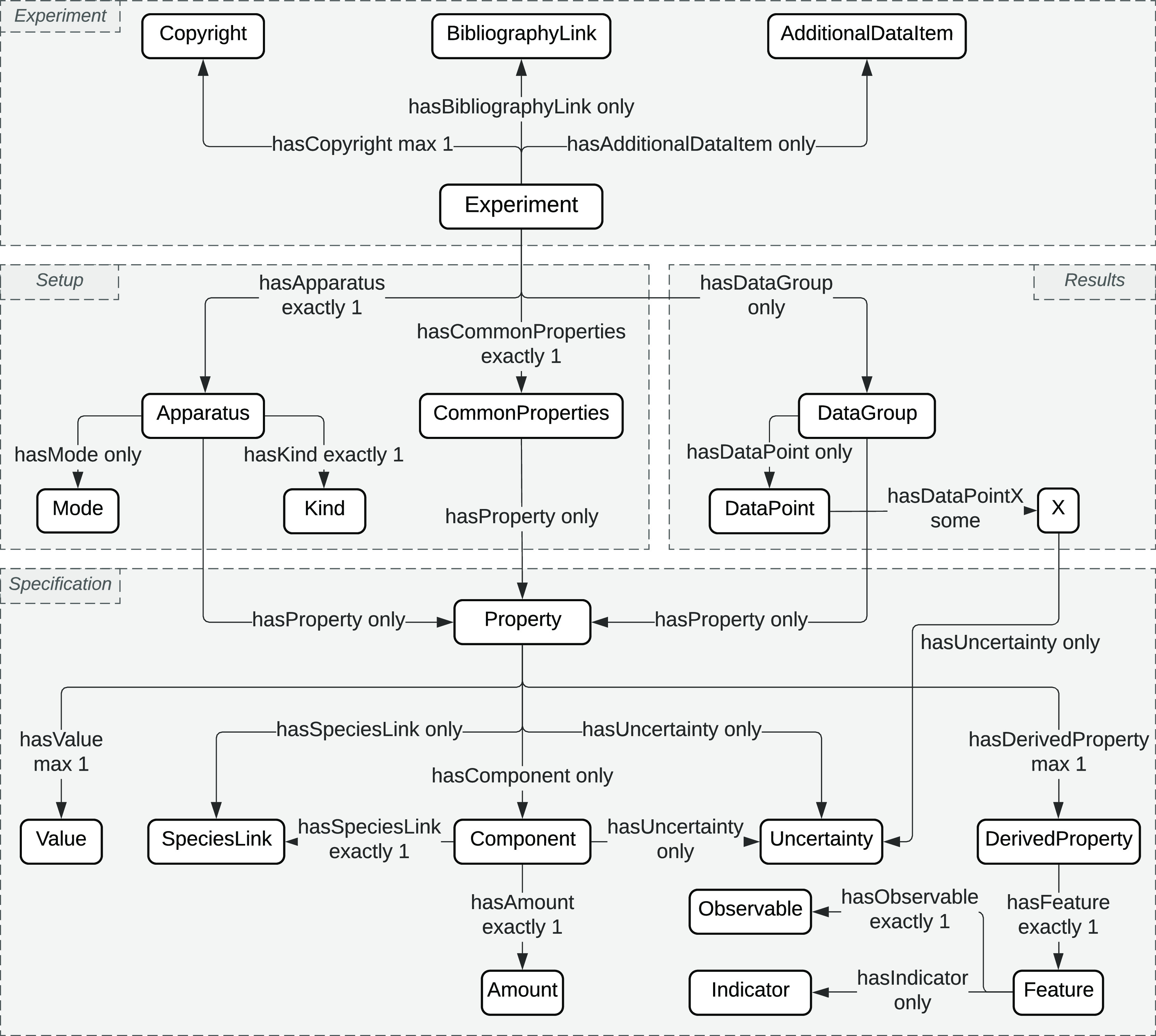
Core concepts and relations of OntoChemExp ontology. This ontology is constructed to represent measurements from combustion experiments. The complete ontology consists of 36 concepts and 60 relations.

Figure 2 illustrates the core concepts and relations defined in OntoChemExp. The conceptual structure is divided into four modules following a heuristic approach:

•Experiment: an experiment refers to the process of observing and measuring characteristics of interest from an energy release chemical process of a mixture of fuel and air. Dependent upon the original source, a set of metadata may be employed to provide more details and more precise identification of an experiment. This metadata includes copyright, bibliography link, and additional data items.
•Setup: the setup outlines the global conditions of an experiment, including the apparatus in which the experiment was conducted and the shared process conditions, forming common properties. The concepts defined in this section are normally left unchanged throughout an experiment.
•Results: experimental results are grouped within data groups that share the same set of independent variables. Within the data groups, individual data points describe each experimental measurement, including independent and dependent variable values that are detailed within *X*.
•Specification: the specification is a shared data structure, supporting both the setup and results modules with an abstract concept property. Property is used to group a wide range of properties. The most straightforward usage is detailing the size of equipment with values. Property is also used to provide information about chemical components with the species described by a species link and an amount, for example, initial composition of the fuel/air mixture. A further use of property is describing derived properties, that include features such as indicators and observables.

As an example, consider the laminar flame speed experiment conducted by Sun et al.^32^ The experiment module contains the metadata related to this experiment, including a bibliography link that points to the publication and an additional data item specifying the description of the nature of the experiment. The setup module documents the apparatus employed, that is, an electrically heated constant-volume cylindrical combustion vessel, as well as the common properties that outline the boundary conditions used in the experiment for all data points, that is, a mixture of PODE_3_/air at atmospheric pressure and an initial temperature of 408 K. This information was classified following the schema in Figure 2 and detailed in the specification module, for example, the mixture of PODE_3_/air is represented by an initial composition property grouping component of individual chemical species with a species link, providing unique species identification and amount indicating concentrations. The results module records the data points collected from the laminar flame speed measurements at equivalence ratios ϕ ranging from 0.7 to 1.6 in the format of data groups. This is also supported by the specification module such that both laminar flame speed measurement and its corresponding equivalence ratio were treated as individual properties that map the human-readable notations (e.g., commonly used mathematical symbol, units, description of this property, etc.) and the numerical values (i.e., *X* as adopted in data points).

**Figure 3:**
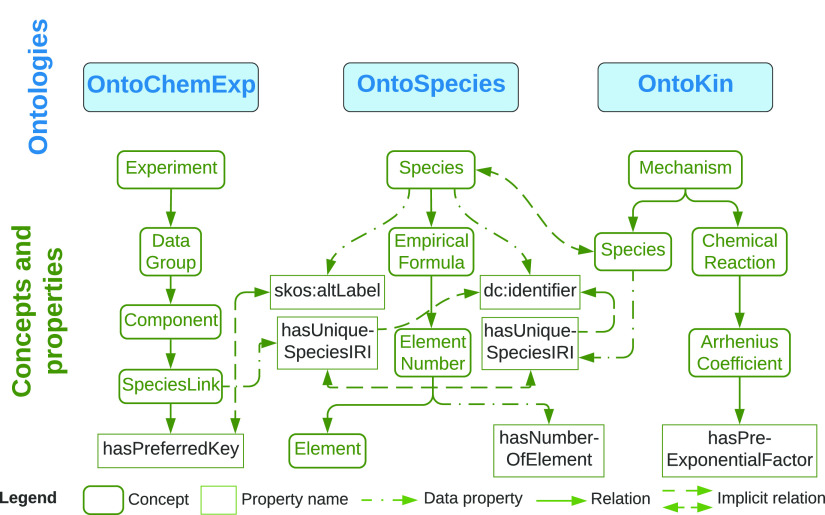
Selected concepts, properties, and relations that demonstrate the links between OntoChemExp, OntoSpecies, and OntoKin ontologies. The main purpose of these links is to enable unique identification of species.

Figure 3 depicts how combustion experiment measurements and chemical mechanisms may be connected. The task of linking species with reactions has already been achieved in previous work,^16^ linking OntoKin with OntoSpecies. This allows the linking of OntoChemExp to both species and reactions via provision of unique species identifiers within OntoSpecies.

Two connections are made between OntoChemExp and the prior ontologies: (1) data property “hasPreferredKey”, equivalent to “skos:altLabel”, that refers to the common name of a species within the community and (2) data property “hasUniqueSpeciesIRI” that directly links to the exact OntoSpecies instance. This design can help resolve inconsistencies between data from different sources through the unique identification of chemical species in OntoSpecies. The importance of the use of this approach is shown by the PODE_3_ demonstration case, whereby poly(oxymethylene) dimethyl ether 3 is denoted differently as PODE_3_ by He et al.,^33^ POMDME_3_ by Sun et al.,^32^ DMM_3_ by Lin et al.,^18^ and OME_3_ by Cai et al.^36^ These ambiguities may be handled by human operators but present a significant challenge to machine interpretability. This challenge is exemplified if PODE_3_ is used as the notation for the initial concentration of ignition delay measurements and POMDME_3_ for the laminar flame speed experiment, whereas DMM_3_ is used in the mechanism. Instead, the linkage is created between ontologies via unique species identification such that one and the same species can be referenced throughout the various stages of calibration, irrespective of the different string labels that may be attached to it (which can be retrieved via SPARQL query) in different contexts. The ontological approach adopted thus facilitates dealing with naming ambiguities of chemical species, allowing for greater interoperability between agents, more comprehensive querying, and many opportunities for semantically driven tasks.

Populating the knowledge graph is managed by a tailor-made tool set developed in this work for generating OntoChemExp-conformant OWL files. The tool set is based on that developed by Farazi et al.^12^ for converting chemical mechanisms to the format of OntoKin. Experimental data related to PODE_3_ were manually constructed in the OWL format of OntoChemExp and then uploaded to the knowledge graph. The knowledge graph was subsequently deployed on an RDF4J (https://rdf4j.org/) triple store, queriable by the SPARQL Protocol and RDF Query Language (SPARQL).

### Agent Integration

**Figure 4:**
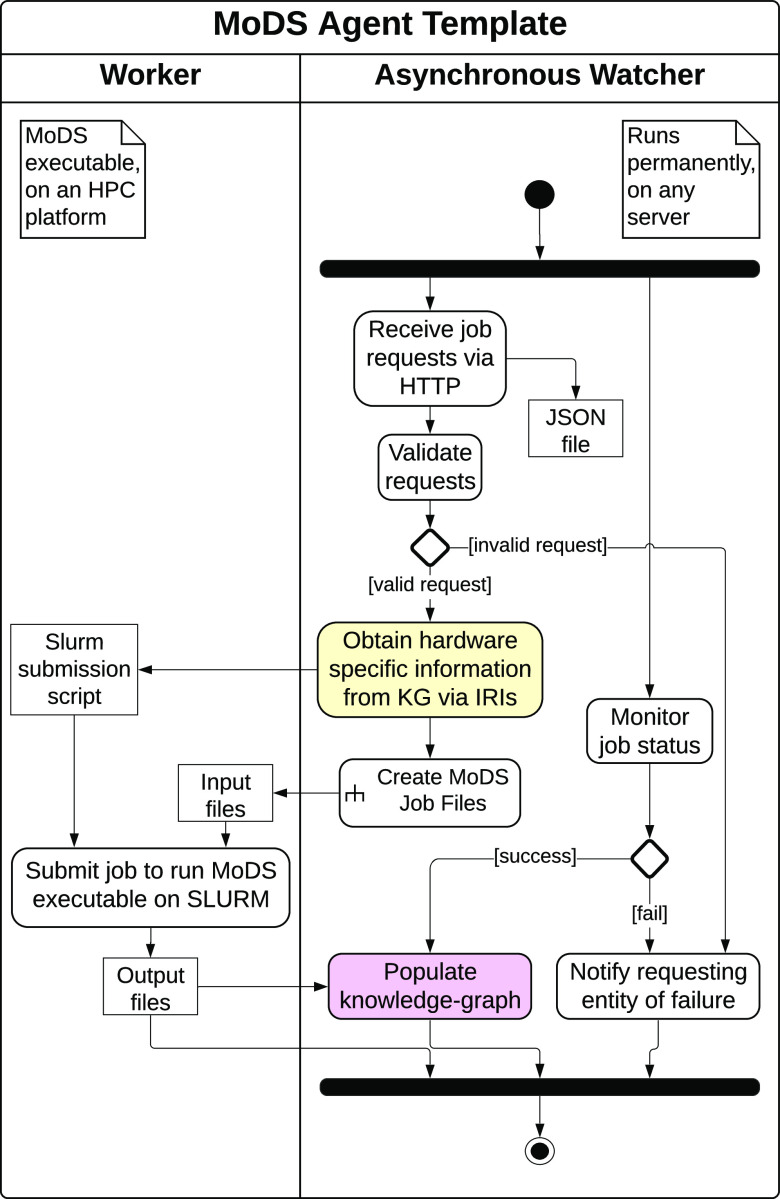
UML activity diagram of a template agent that enables MoDS jobs to be executed asynchronously on an HPC platform upon HTTP requests. The same design is followed by all MoDS wrapper agents, distinguished by different activate nodes for job file creation. The yellow shaded action indicates the data retrieving operation of agents over the knowledge graph, whereas magenta refers to the knowledge graph populating operation.

The framework detailed and developed in this work is intended to act as an agent within the JPS ecosystem. It is structured as an instantiation of the agent template proposed by Mosbach et al.^17^ A unified modeling language (UML) activity diagram of the agent is provided in Figure 4, with the agent template surrounding the model development suite (MoDS)^48^ software package. MoDS is an integration of multiple tools developed for various generic model development tasks, such as parameter estimation,^49^ surrogate model creation,^50,51^ and experimental design.^52^

Detailed documentation of the agent template is provided by Mosbach et al.,^17^ so only the changes from the template design will be detailed. One such change is the addition of a validation step for received job requests. This is added in order to improve the robustness of the agent. The validation step ensures that the job request to the sensitivity analysis agent contains the IRIs that point to the chemical model and the experiment data against which the sensitivity analysis is conducted. In addition, the job request to the mechanism calibration agent must contain the IRIs provided by either the user or the sensitivity analysis agent pointing to the active parameters to be optimized. The second change has been made to merge the process of querying executable-specific information from the knowledge graph with the process of creating job files. This was implemented to accommodate different types of jobs being requested due to the integration of MoDS with multiple tools and its capability as a generic model development tool.^49,52,53^ This results in an agent capable of automatically generating specific job files corresponding to supplied job requests. Once passed the request validation, the agent will query model parameters and experiment process conditions in the case of sensitivity analysis. By contrast, for mechanism calibration, additional queries are made for the list of active parameters and experimental responses.

The MoDS agent is designed to accept a target mechanism and experimental results under a range of process conditions and to perform parameter estimation for the target mechanism. To achieve this, the agent performs simulations with the experimental conditions and adjusts parameters within the target mechanism to replicate the experimental responses. The responses cover different simulation tasks which are performed in two different software packages, necessitating the generation of individual executable models. The software packages were *k*inetics^37^ for ignition delay time simulation and Cantera^38^ for laminar flame speed simulation.

**Figure 5:**
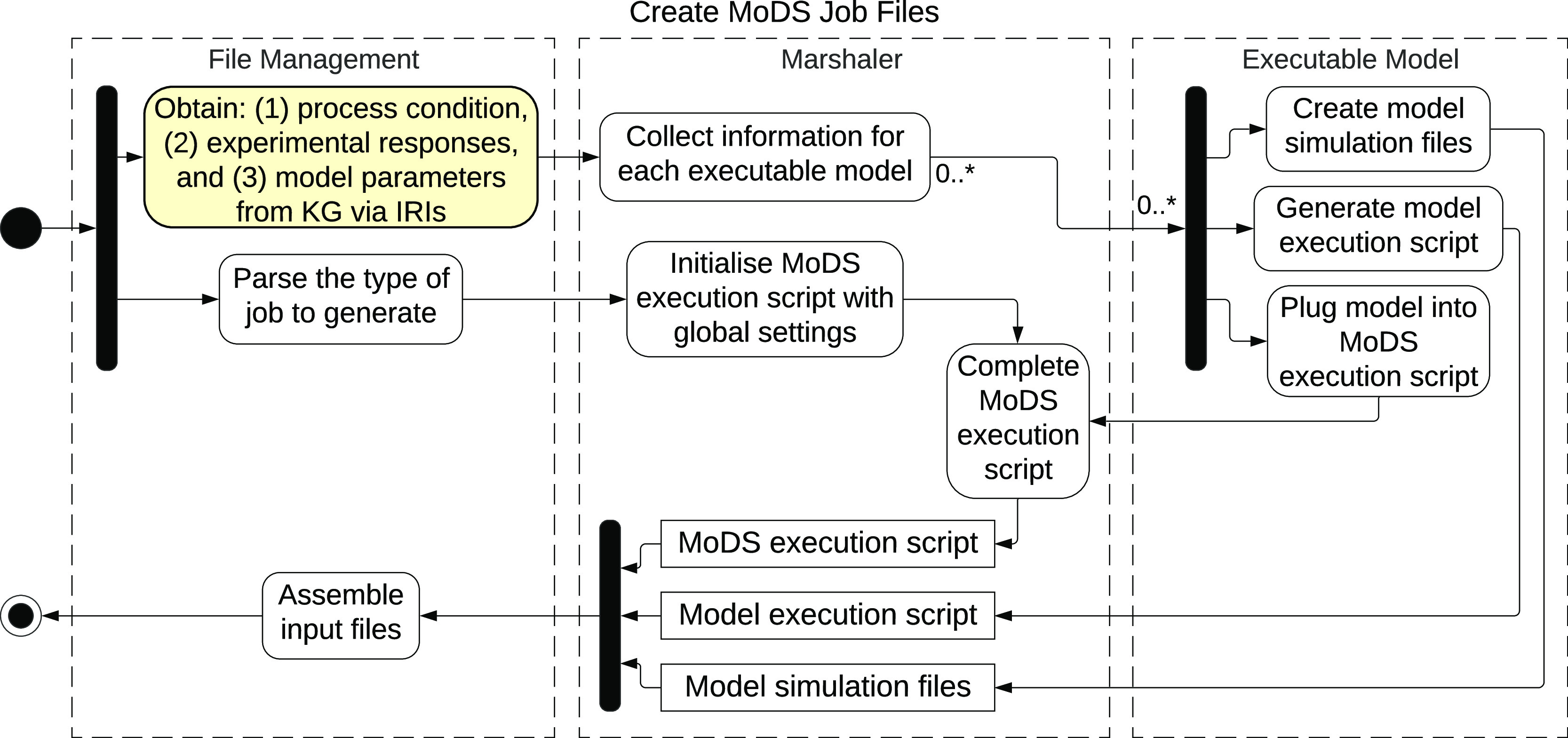
Workflow of the process of creating requested job files. The whole process corresponds to the activity node in Figure 4.

Figure 5 illustrates the automated process of generating MoDS job files. This process is structured in three layers: the file management center communicates the input and output, a marshaler collects all information required by the MoDS executable, and finally, the layer that manages the individual executable models. In the file management center, the process starts by querying the knowledge graph for information related to each experiment response. This information is then passed to the marshaler to allocate executable models for simulating each response. The simulation files and execution script required for the selected models are then generated in the executable model layer and sent back to the marshaler.

At the same time, the type of job requested is parsed in the file management center and passed to the marshaler to initialize a MoDS execution script with predefined global settings. This script is connected to the selected models generated by the executable model layer. All generated files are then assembled in the file management center and transferred to an HPC platform.

The first two stages in the automated mechanism calibration are performed by two MoDS template agents: MoDSSensAnaAgent for sensitivity analysis and MoDSMechCalibAgent for mechanism calibration. Three parameters are currently available for the sensitivity analysis: magnitude of the relative perturbation, the type of overall sensitivity (maximum absolute value or average absolute value), and the number of reactions to be optimized.

For the mechanism calibration, the parameters are made available in two folds: the global settings for the algorithms used in the MoDS job and the calibration objective parameters. For the algorithms, the total number of Sobol points to be generated and the termination tolerance of the Hooke–Jeeves algorithm can be specified by the user. For the calibration objective parameters, two parameters are available: the weighting in the objective function and the response scaling type (logarithmic or linear). Provision is also made for users to supply their own list of reactions to guarantee their inclusion in the calibration process.

A coordination agent manages the interactions between the MoDSSensAnaAgent and MoDSMechCalibAgent with the knowledge graph. These three agents form the overall automated mechanism calibration agent, AutoMechCalibAgent.

**Figure 6:**
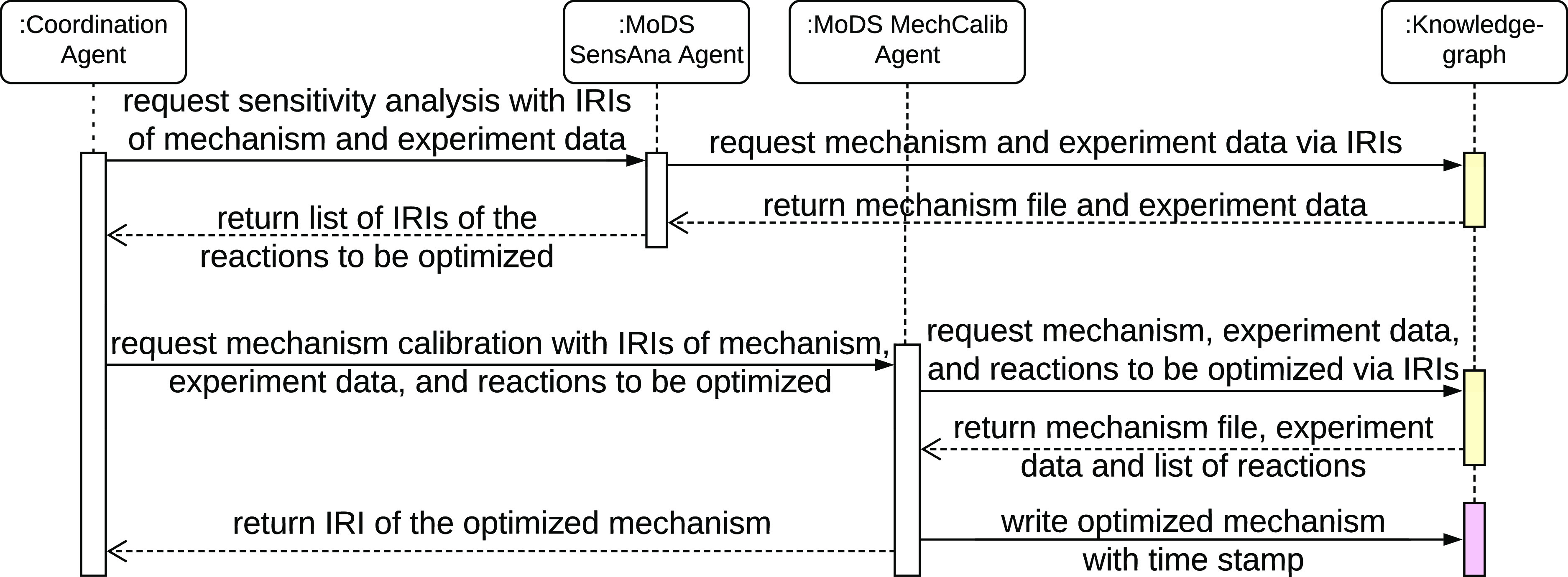
UML sequence diagram of the automated mechanism calibration process that captures the interaction between the different agents and the knowledge graph. Actions where the agent retrieves data from the knowledge graph are shaded in yellow and those where the agent populates the knowledge graph in magenta.

Figure 6 illustrates the UML sequence diagram of the AutoMechCalibAgent as a five-step process. Initially, the coordination agent validates the job request and invokes the MoDSSensAnaAgent for a sensitivity analysis via IRIs. Second, the MoDSSensAnaAgent communicates with the knowledge graph via IRIs to obtain the chemical model to be calibrated and the process conditions over which the sensitivity analysis is to be conducted. After the sensitivity analysis, the MoDSSensAnaAgent returns the list of IRIs of the identified reactions to the coordination agent. Third, the coordination agent requests MoDSMechCalibAgent for a mechanism calibration job with the reactions identified. Fourth, the MoDSMechCalibAgent carries out the calibration. Global search and local optimization are used with experimental data retrieved from the knowledge graph. As the final step, the MoDSMechCalibAgent populates the knowledge graph with the calibrated mechanism and returns its IRI to the coordination agent.

The process relied upon linked ontologies. These provided the connection between combustion experiments (OntoChemExp) and kinetic mechanisms (OntoKin) via unique identification of chemical species (OntoSpecies).

## Results and Discussion

### Sensitivity Study

Prior to the automated calibration task, a sensitivity study was performed to assess the effect of the relative perturbation size used during the reaction selection sensitivity analysis in the automated calibration process. This sensitivity study was performed using the MoDSSensAnaAgent developed. The study involved 21 relative perturbation sizes for the finite difference approximations (*r* in eq 3) of the derivatives required for the sensitivity coefficients (1 × 10^–*n*^, 2 × 10^–*n*^, and 5 × 10^–*n*^; *n* = 1, ..., 7) and assess the sensitivities for both ignition delay times and laminar flame speeds to the Arrhenius pre-exponential factor for all 215 reactions. For every reaction, a normalized sensitivity coefficient was computed for all 73 experimental conditions used for the calibration process. The maximum absolute value form of the sensitivity coefficient was used, and the reactions ranked based on this value to determine their relative importance. We present the 10 reactions with the greatest absolute sensitivity coefficients.

**Figure 7:**
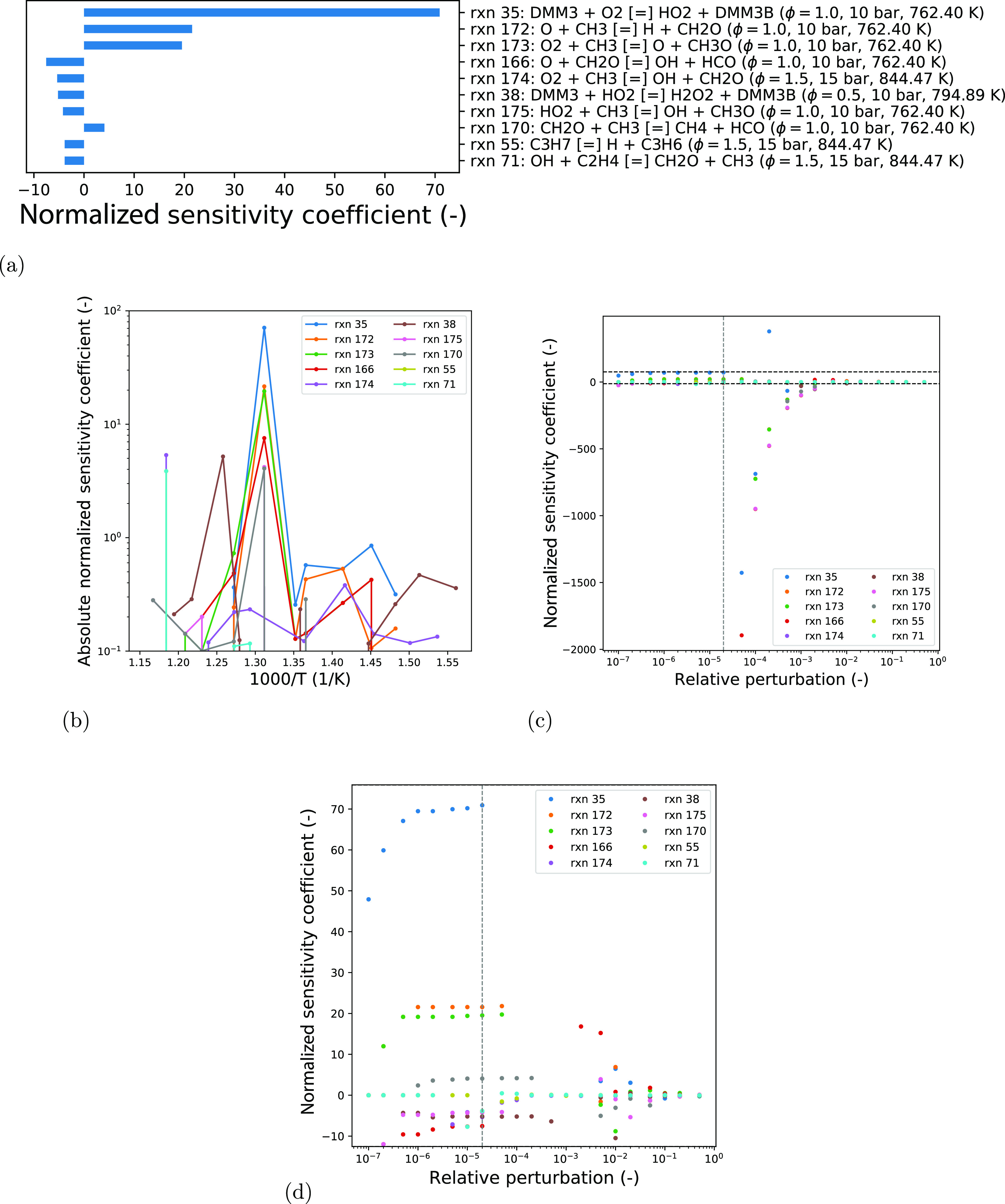
Sensitivity analysis of the ignition delay times with respect to Arrhenius pre-exponential factors in the starting mechanism. The list of reactions is selected based on the maximum value of its sensitivities across all considered points in an experimental condition space with a relative perturbation of 2 × 10^–5^. (a) Selected 10 most sensitive reactions using a relative perturbation of 2 × 10^–5^, the labeled condition for each reaction corresponds to that at which the peak value in (b) was obtained. (b) Sensitivities as a function of temperature. (c) Sensitivities as a function of relative perturbation. (d) Same as (c) but for a smaller range of sensitivities, as indicated by the horizontal lines.

Figure 7 presents the ignition delay time results of the sensitivity study. The values of the sensitivity coefficients at their maximum absolute value are shown in Figure 7a along with the conditions for each case. The effect of temperature on the sensitivities at a fixed equivalence ratio and pressure is shown in Figure 7b. As demonstrated by the variability in normalized sensitivity coefficients to changing conditions, different sets of active parameters can be identified. This includes in particular, different active parameters for low- *versus* high-temperature regions. It is thus necessary to assess reaction importance through a global perspective. Figure 7c shows the stability of the sensitivity coefficients over the range of relative perturbation sizes investigated. The horizontal dashed lines bound the region that clearly illustrates the variation for all reactions, as magnified and presented in Figure 7d. It can be seen that the model, like most combustion models, is highly nonlinear. The selection of suitable relative perturbation sizes is thus in favor of small values, where the model behaves in a relatively linear fashion for most of the reactions. The peaks in Figure 7b correspond to the sensitivity coefficients used for ranking the reactions, matching the values at the vertical dotted line in Figure 7d and those presented in Figure 7a.

This study identified two reactions from the PODE_3_ submechanism, reactions 35 and 38. Reaction 35 belongs to the first O_2_ addition reaction class. This class is suggested by Ren et al.^35^ as a good choice for calibration as ignition delay times are usually sensitive to this class of reactions at low initial temperatures. Reaction 38 involves H-abstraction by HO_2_ radicals, shown to increase the fuel reactivity, thus important to PODE_3_ combustion.^36^

**Figure 8:**
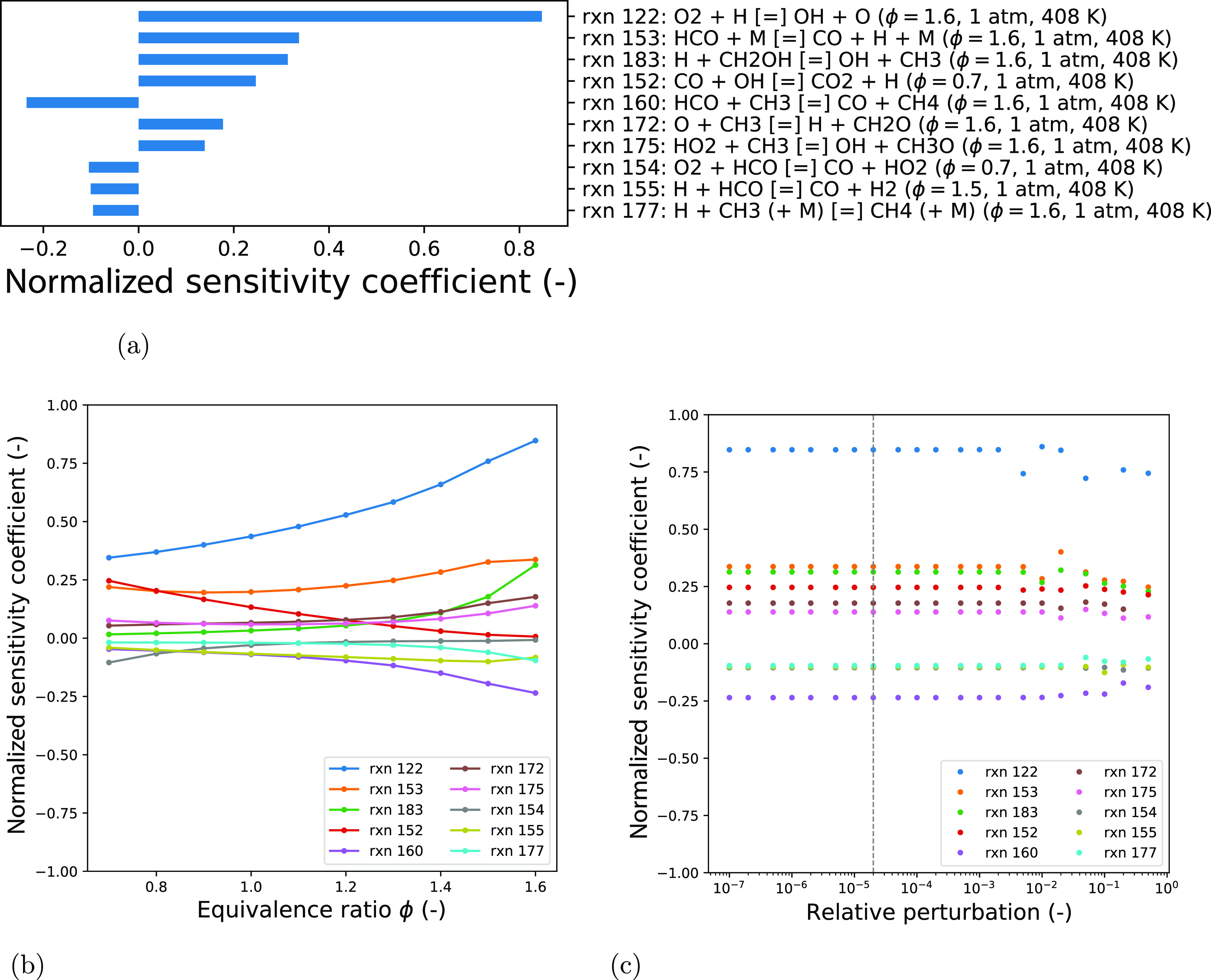
Sensitivity analysis of the laminar flame speed with respect to Arrhenius pre-exponential factors in the starting mechanism. The list of reactions is selected based on the maximum value of its sensitivities across all considered points in the experimental condition space with a relative perturbation of 2 × 10^–5^. (a) Selected 10 most sensitive reactions using a relative perturbation of 2 × 10^–5^. (b) Sensitivities as a function of the equivalence ratio. (c) Sensitivities as a function of the relative perturbation.

Figure 8 presents the sensitivity analysis results for laminar flame speeds. The structure remains similar to that of Figure 7 but with Figure 8b showing the effect of the equivalence ratio rather than temperature.

Reaction 122 was found to have the greatest influence on the laminar flame speed. This is a chain-branching step O_2_ + H ↔ OH + O and is expected to have a significant effect on the laminar flame speed^54^ chap. 8. Other reactions identified mostly involve small species and radicals, which govern a large part of the heat release (e.g., CO + OH ↔ CO_2_ + H).^55^

The reactions identified for both laminar flame speed and ignition delay times fall in line with those selected while Lin et al.^18^ constructing the reduced mechanism. The mechanism was developed using a decoupling methodology, separating the mechanism detail into three levels: detailed for H_2_/CO/C_1_, reduced for C_2_–C_3_, and skeletal for C_4_–C_N_. As appearing in the list of reactions found to be sensitive for laminar flame speed, most of them are in the detailed H_2_/CO/C_1_ submechanism, representing a high-temperature combustion process. While for ignition delay times, the most sensitive reaction is from a skeletal structure for C_4_–C_N_, representing a low-temperature combustion process.

Following this analysis, a relative perturbation size of 2 × 10^–5^ was chosen for the calibration process. This value is selected to act as a trade-off between numerical errors and the onset of nonlinearities, given that larger sizes present systematic changes in sensitivity as a function of perturbation, specifically for ignition delay times, whereas smaller values increase the likelihood of problems due to rounding errors.

### Mechanism Calibration

In parameter estimation tasks, deciding the number of parameters to include within the estimation task requires consideration of the trade-offs between precision and tractability. For this calibration case, the reactions to optimize have been set as the top 10 reactions identified for ignition delay times and laminar flame speeds by the MoDSSensAnaAgent. This resulted in a total of 18 reactions to optimize due to reactions appearing in both sensitivity analyses.

Another trade-off requiring consideration is that of the weighting between ignition delay times and laminar flame speeds. In many cases, differing quantities of experimental data are available and there may exist differences in users’ preference in weightings. The weighting of the two is handled by the value of α in the objective function of the calibration process (i.e., eq 6). In this case, there are 63 ignition delay time experimental data points and 10 laminar flame speeds. Correcting this imbalance in the number of experimental results forms a natural starting point for selecting a value for α, and so values of α in the range of 6.3–1 were investigated. This range is intended to cover values that offer a good balance between the two responses and prevent the domination of ignition delay times for the calibration.

The calibration routine seeks to optimize the values of the pre-exponential factors for the target reactions. The range selected for the task was 10^–2^ to 10^2^ times the original value. This is a relatively large range in relation to physical uncertainties; however, this is a reduced mechanism and individual reactions must not be misinterpreted as elementary, physical reactions. The process began with Sobol sampling within the selected range of values; 10^4^ logarithmic, evenly distributed points were used to determine three starting points for the optimization routine that displayed the lowest values of the objective function.

Following from the sampling stage, a Hooke–Jeeves optimization routine is performed. The routine was performed with 400 iterations and a termination step size of 0.001, with an initial step size of 0.2 and step size reduction factor of 0.5. The results of the sampling and optimization stages are presented in Table 2.

**Table 2:** Objective Function of Global Search and Local Optimization Results of the Starting Mechanism^a^

ratio α	best Sobol	H–J	2nd Sobol	H–J	3rd Sobol	H–J
6.3	4446	**659**	5153	1375	5356	1263
4.98	4170	**657**	4697	1378	4788	895
3.65	3892	**710**	4216	712	4238	1398
2.33	3617	896	3648	**714**	3783	1373
1	3075	654	3165	**653**	3324	1370

a Best three Sobol points from global search, that is, the three Sobol points with the smallest objective function values, were chosen for further local optimization with a Hooke–Jeeves (H–J) algorithm. Each of these Sobol points represents a combination of the active parameters sampled within the selected range. Values in bold face indicate the best-performing mechanism for each response ratio and are chosen as the starting mechanism for the next iteration of calibration.

Prior to the sampling stage, the scaled sum-of-squares-error value was found to be 14554 for ignition delay times and 774 for laminar flame speeds, resulting in objective function values of 19430 and 15328 for α values of 6.3 and 1, respectively. This indicates the value of performing the initial Sobol sampling stage, with a significant improvement in the objective function being achieved prior to any optimization. This is particularly valuable as the Hooke–Jeeves algorithm performs local search, significantly benefiting from a good initial point.

The optimization stage is further seen to be providing an improvement in the objective function, significantly reducing its value from the sampling stage. The results of the optimization stage are comparable, in the sense of having the same order of magnitude, with those of Lin et al.,^18^ which achieved an objective function value of 140 with an α value of 1.

Although variation is seen in the objective function values after the sampling stage in response to changing α values, the same change is not observed after the optimization stage. This is a result of the contributions to the objective function from the laminar flame speeds becoming very small after optimization. The laminar flame speed is largely governed by small-molecule oxidation which remains a detailed submechanism within the decoupling methodology adopted in the Lin et al.^18^ mechanism. This suggests that a better fit would be expected for both the initial mechanism and calibrated mechanism for laminar flame speeds than ignition delay times.

During the initial sensitivity analysis, the mechanism is unable to accurately reproduce the combustion characteristics. This suggests that reaction selection at this stage may be premature and may not select all reactions that are of the most importance locally to the optimum fitting. Additional reactions may also only become important after the rates of the initially identified reactions are closer to their optimum values. For these reasons, a further iteration of the calibration algorithm is necessary, which is consistent with the recommendation by Frenklach.^39^

### Second Iteration

A second calibration was performed on the best-performing mechanism for each value of α. The ontological structure of the framework aided in this process, allowing for the task to be completed by the passing of the IRIs for the experiments and calibrated mechanisms from the last iteration to the AutoMechCalibAgent agent, with the rest of the configuration identical to the first iteration.

After validating the job request, the coordination agent requested the MoDSSensAnaAgent to perform a sensitivity analysis to identify the key reactions. Since the sensitivities depend not only on the conditions but also on the model parameters, the active parameters identified are different. The list of IRIs for the updated active parameters was then added to the original job request by the coordination agent and passed to the MoDSMechCalibAgent. A mechanism calibration was then performed to optimize the mechanism. All other settings for the sensitivity analysis (i.e., relative perturbation size, the type of overall sensitivity, and the number of reactions to be optimized) and mechanism calibration (i.e., the global settings for the algorithms and the calibration objective parameters) were left unchanged. The results after both the sampling and calibration stages are summarized in Table 3.

**Table 3:** Objective Function Values after Sampling and Optimization for the Best-Performing Mechanisms Selected from the First Iteration of Mechanism Calibration for Each α Value^a^

ratio α	best Sobol	H–J	2nd Sobol	H–J	3rd Sobol	H–J
6.3	605	278	627	296	651	**260**
4.98	375	89	505	**79**	507	187
3.65	459	**243**	500	259	552	253
2.33	571	38	658	133	702	127
1	355	151	376	**133**	377	156

a All mechanisms showed significant improvement in this iteration of calibration, with the best-performing mechanism underlined.

After the calibration stage, the best-performing mechanism was found with an α value of 2.33. The objective value of the calibrated mechanism (Φ = 38) is found to show a 79% decrease compared to that of the Lin et al.^18^ mechanism (Φ = 181) when the same α values of 2.33 are used in the current definition of the objective function (eq 6).

**Figure 9:**
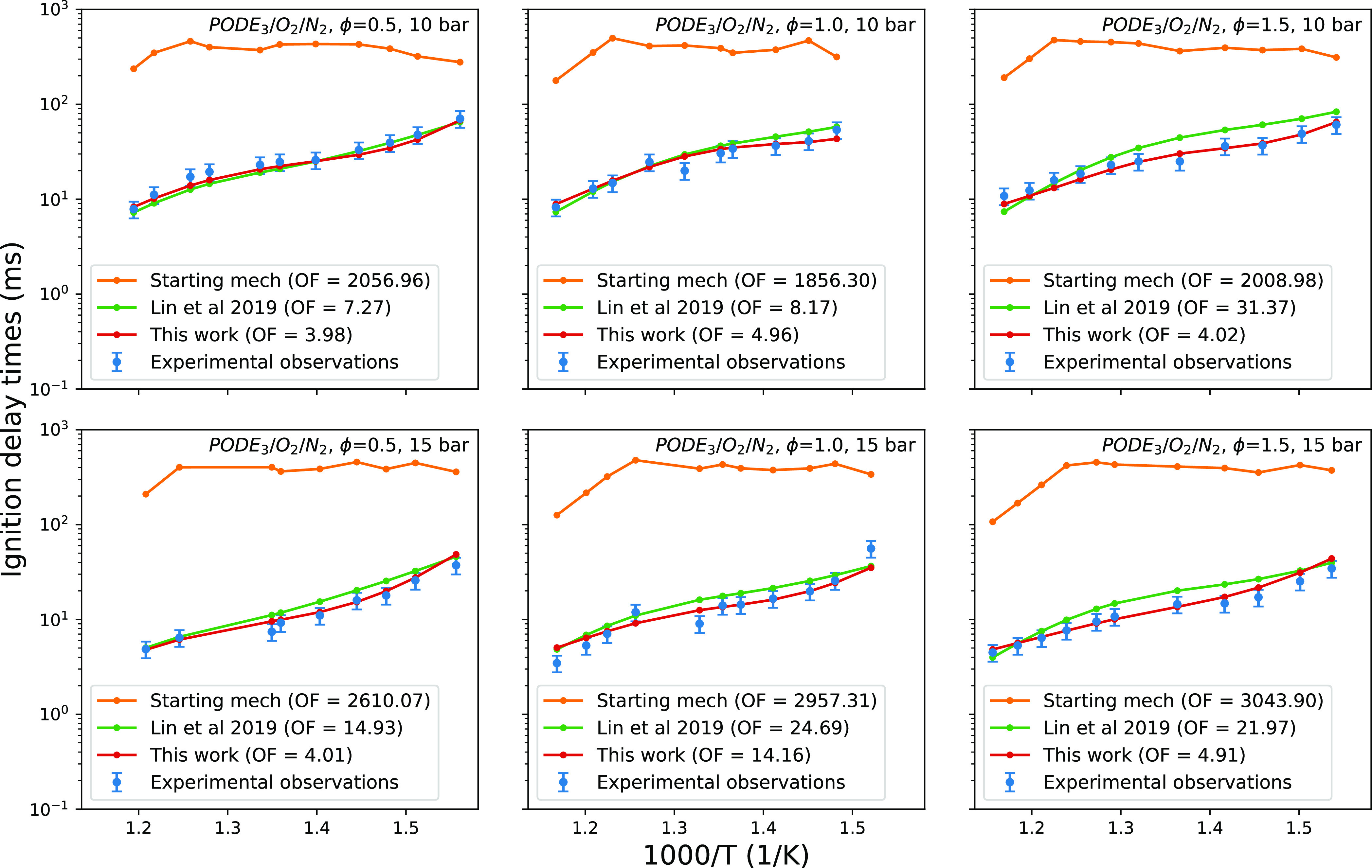
Comparison of the mechanisms from^18^ and the AutoMechCalibAgent agent (this work) at simulating ignition delay times (maximum rate of pressure increase ignition criterion) of PODE_3_/O_2_/N_2_ mixtures at three equivalence ratios.^33^ The model performance is displayed as the ignition delay time contribution to the objective function. As per the experimental results, the oxidizer used in this study has different compositions: (1) ϕ = 0.5, O_2_/N_2_ = 1:8; (2) ϕ = 1.0, O_2_/N_2_ = 1:15; (3) ϕ = 1.5, O_2_/N_2_ = 1:20.

**Figure 10:**
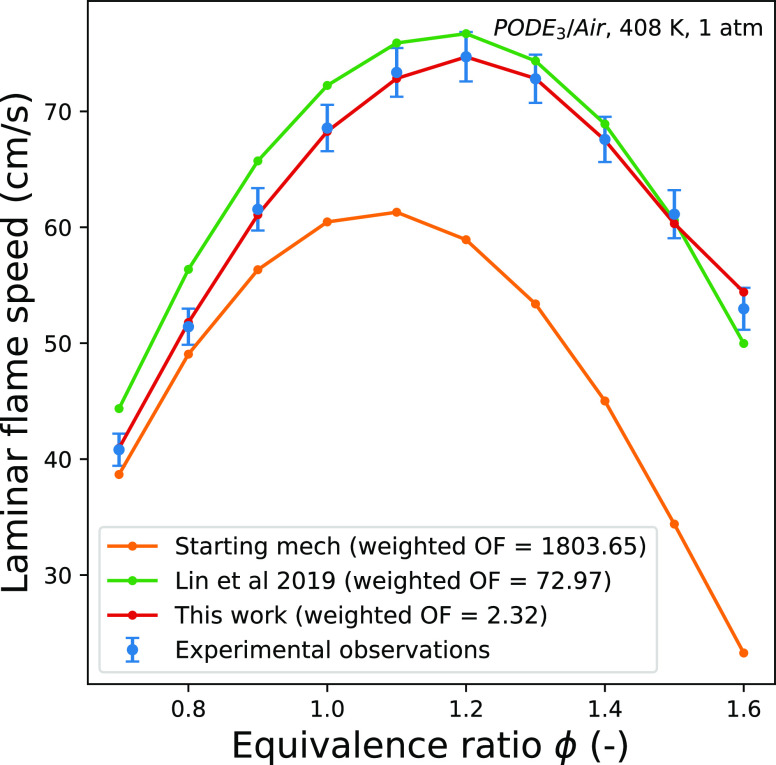
Comparison between the model from^18^ and the AutoMechCalibAgent agent (this work) on simulating laminar flame speed of PODE_3_/air mixtures at atmospheric pressure and an initial temperature of 408 K.^32^ The model performance is displayed as the value of the laminar flame speed contribution to the objective function with an α value of 2.33.

The performance of the mechanism of Lin et al.^18^ (manual calibration) and the mechanism of this work (automated calibration) is compared in Figures 9 and 10. The automatically calibrated mechanism shows a good fit to the experimental data in all cases. It should be noted that in the original paper of Lin et al.,^18^ a temperature rise of the 400 K criterion was used for calibrating and assessing their model against ignition delay times, yielding an objective function value of 121 according to eq 6. In this work, a maximum rate of the pressure-increase criterion is used when comparing the models’ performance in Figure 9, resulting in an objective function value of 108 for the Lin et al.^18^ model. This change of ignition criterion brings it in line with that used for the experimental results, and we note that this represents an improvement for the Lin et al.^18^ model.

The negative temperature coefficient (NTC) behavior of the fuel is captured in both the mechanism of this work and that of Lin et al.^18^ In Lin et al.,^18^ it is claimed that capturing the NTC region is achieved through optimization of the isomerization reaction DMM_3_BO_2_ ↔ DMM_3_OOH_35_.·In this work, this reaction was not identified as important and so was not calibrated, instead remaining at the same value as used by He et al.,^33^ where no apparent NTC region was captured.

It is believed the capturing of the NTC behavior in this work is a result of the sensitivity analysis identifying reactions of importance in the intermediate-temperature regime (around 770 K), corresponding to the NTC region. This effect may be seen in Figure 7b, in which the majority of the sensitivities show a peak in the intermediate-temperature region.

Table 4 summarizes the changes made to the Arrhenius pre-exponential factors during the calibration process. The range of adjustment for the rate parameters during the calibration process was 10^–4^ to 10^4^. While this may be considered a wide range, we note that other studies dealing with reduced mechanisms report similar orders of adjustment, such as Lin et al.^18^ and Chang et al.^56^ while calibrating mechanisms constructed with decoupling methodologies. In contrast to Lin et al.^18^ and Chang et al.,^56^ however, the reduced mechanism in this work is optimized as a whole to fit the provided experimental data. It is further noted that even more complete PODE_3_ mechanisms, such as that of Ren et al.,^35^ modify the pre-exponential factors by an order of magnitude during calibration. This is to balance necessary levels of adjustment against unnecessarily large search spaces.

**Table 4:** Summary of the Calibrated Arrhenius Pre-Exponential Factors^a^

reaction	equation	original A factor	First iteration	Second iteration
35	DMM_3_ + O_2_ ↔ HO_2_ + DMM_3_B	6.66 × 10^6^	6.66 × 10^8†^	1.37 × 10^10†^
36	DMM_3_ + OH ↔ H_2_O + DMM_3_B	3.79 × 10^–2^		4.22 × 10^–4†^
37	DMM_3_ + H → H_2_ + DMM_3_B	7.40 × 10^6^		5.05 × 10^5†^
38	DMM_3_ + HO_2_ ↔ H_2_O_2_ + DMM_3_B	4.00 × 10^7^	2.25 × 10^9†^	1.24 × 10^9†^
55	C_3_H_7_ ↔ H + C_3_H_6_	1.25 × 10^14^	1.08 × 10^13†^	
71	OH + C_2_H_4_ ↔ CH_2_O + CH_3_	1.00 × 10^8^	3.23 × 10^6†^	
122	O_2_ + H ↔ OH + O	1.04 × 10^8^	1.04 × 10^10‡^	1.72 × 10^11‡^
124	H_2_ + O ↔ OH + H	8.79 × 10^8^		1.35 × 10^8‡^
126	OH ↔ H_2_O + O	3.34 × 10^–2^		4.19 × 10^–3‡^
152	CO + OH ↔ CO_2_ + H	2.23 × 10^–1^	6.22 × 10^–1‡^	1.16 × 10^1‡^
153	HCO + M ↔ CO + H + M	5.75 × 10^5^	2.13 × 10^5‡^	8.30 × 10^5‡^
154	O_2_ + HCO ↔ CO + HO_2_	7.58 × 10^6^	2.02 × 10^6‡^	
155	H + HCO ↔ CO + H_2_	7.23 × 10^7^	4.63 × 10^9‡^	1.04 × 10^8‡^
160	HCO + CH_3_ ↔ CO + CH_4_	1.20 × 10^8^	9.40 × 10^9‡^	3.80 × 10^10†‡^
166	O + CH_2_O ↔ OH + HCO	1.81 × 10^7^	1.21 × 10^7†^	
167	OH + CH_2_O ↔ H_2_O + HCO	3.43 × 10^3^		6.79 × 10^3†^
170	CH_2_O + CH_3_ ↔ CH_4_ + HCO	3.64 × 10^–12^	5.78 × 10^–11†^	5.38 × 10^–9†^
172	O + CH_3_ ↔ H + CH_2_O	8.43 × 10^7^	8.43 × 10^5†‡^	
173	O_2_ + CH_3_ ↔ O + CH_3_O	1.99 × 10^12^	1.99 × 10^14†^	7.25 × 10^14†^
174	O_2_ + CH_3_ ↔ OH + CH_2_O	3.74 × 10^5^	6.56 × 10^6†^	9.33 × 10^4†^
175	HO_2_ + CH_3_ ↔ OH + CH_3_O	1.00 × 10^6^	1.00 × 10^8†‡^	
177	H + CH_3_ (+M) ↔ CH_4_ (+M)	1.27 × 10^10^	1.03 × 10^9‡^	
180	OH + CH_4_ ↔ H_2_O + CH_3_	5.72 × 10^0^		5.03 × 10^2‡^
183	H + CH_2_OH ↔ OH + CH_3_	9.64 × 10^7^	3.30 × 10^9‡^	3.78 × 10^9‡^
186	O_2_ + CH_2_OH ↔ HO_2_ + CH_2_O	2.41 × 10^8^		2.41 × 10^10‡^
193	CH_3_O + M ↔ H + CH_2_O + M	8.30 × 10^11^		2.26 × 10^12†^

a Omitted values imply that a reaction rate is unchanged. The unit of the pre-exponential factors is m^3^ mol^–1^ s^–1^ or s^–1^ for two and one reactant, respectively. The indexes of reactions follow the labels generated while converting the mechanism from the CHEMKIN to OntoKin format. Reactions identified as sensitive for different response are denoted as † for ignition delay time and ‡ for laminar flame speed. Note that PODE_3_ is denoted as DMM_3_.

Two additional H-abstraction reactions from the PODE_3_ submechanism and a total of eight reactions were identified by the second calibration iteration that were not identified by the first. The second iteration fully captures the governing reactions of the low-temperature combustion process, as found to be important for modeling ignition delay times.^18^ Thus, the substantial improvement of model performance found in the second iteration is not surprising.

Having been optimized in two stages against 73 data points, using 18 and 19 active parameters, respectively, the model is seen to be agree well with the available data points, capturing major trends without incorporating noise present in the experimental data. Although the model performance is not assessed for process conditions outside the range used in this study, one of the aims of the knowledge graph-based approach is that the calibration can be easily repeated once new data are made available.

The calibrated mechanism is available in CHEMKIN format in the Supporting Information, also available at https://doi.org/10.17863/CAM.59826. It should be noted that the chemical model should only be used as a whole, and individual rate parameters should not be used outside of this model. This particularly applies to reactions whose rates are well established in the literature, with relatively narrow uncertainty bounds. For such reactions, the adjusted rates as part of a reduced and calibrated mechanism such as the one in this work may well be unphysical in the sense that they have been adjusted well beyond their established uncertainty bounds. One approach to alleviate this problem, as taken by Lin et al.,^18^ is to calibrate only those reactions whose rates are not well known (here, the PODE_3_ submechanism), while leaving the ones with well-known rates unchanged (e.g., the core C_1_–C_3_ chemistry). A more general approach is to calibrate the chosen reactions within their respective ranges of uncertainty (see, e.g., Sheen and Wang^46^). As the focus of the present work is to take the first steps in the development of the knowledge graph and agent infrastructure, we have omitted such treatments for simplicity at this stage. Taking uncertainties into account is, however, a natural next step, and this is in fact work already in progress.

## Conclusions

In this work, a knowledge graph-based automatic mechanism calibration framework is developed. This acts as an extension to the world avatar (theworldavatar.com), a dynamic knowledge graph ecosystem. All components developed in this work are standardized and modularized to allow them to easily integrate with the wider knowledge graph infrastructure.

For the development process, an ontology, OntoChemExp, was created. OntoChemExp provides an ontological description of combustion experiments and allows for linking these to existing ontologies for reaction mechanisms and chemical species, semantically enriching the description of experiments and drawing links between mechanisms for combustion processes and their experimental validation.

Another contribution of this work is a set of agents for coupled sensitivity analysis and mechanism calibration. These are based on a standardized JPS agent template and are designed to employ generic model development software.

As a demonstration of these technologies, a case study was used of a reduced PODE_3_ combustion mechanism. It was found that two iterations of the coupled agent process were required to sufficiently optimize this mechanism due to its initially poor fitting. The initial iteration brought the calibration objective to a value of a similar order to that of the manual calibration of Lin et al.,^18^ while the second iteration reduced its value to 21% of the manual calibration value. This represents the development of a model which fits the data significantly more accurately, as measured by the stated objective function in a short time span. Should multiple iterations be performed, users need simply provide the IRIs of experimental data and the mechanism from the previous iteration.

This work has demonstrated how knowledge graph technology can be used to improve the data provenance and mechanism calibration. The creation of the OntoChemExp ontology represents a step toward greater provenance determination for combustion mechanisms as mechanisms may be related to the experimental results used in their calibration. The development of an automated mechanism calibration framework addresses another necessary development for the community of developing tools toward coupled and automated sensitivity analysis and calibration of existing combustion mechanisms. At present, the framework is intended to act as a tool for facilitating and enriching the process of mechanism calibration, but the design is intended to allow for future development and refinement to open up new applications made possible by the linked nature and interoperability of knowledge graph representation.

## Supporting Information Available

Chemical mechanism of PODE oxidation in the CHEMKIN format (ZIP)

## Abbreviations

RMG: reaction mechanism generator
GNN: graph neural network
DFT: density functional theory
PrIMe: Process Informatics Model
XML: eXtensible Markup Language
AI: artificial intelligence
OWL: web ontology language
CML: chemical markup language
PODE: poly(oxymethylene) dimethyl ether
JPS: J-Park simulator
IRI: internationalized resource identifier
PRF: primary reference fuel
CLARA: class-based automatic reaction alternator
RCM: rapid compression machine
UF: uncertainty factors
POMDME: poly(oxymethylene)dimethyl ether 3
DMM: dimethoxymethane
OME: oxymethylene ether
SPARQL: SPARQL Protocol and RDF query language
UML: unified modeling language
MoDS: model development suite
NTC: negative temperature coefficient
